# Genomic and Immune Approach in Platinum Refractory HPV-Negative Head and Neck Squamous Cell Carcinoma Patients Treated with Immunotherapy: A Novel Combined Profile

**DOI:** 10.3390/biomedicines10112732

**Published:** 2022-10-28

**Authors:** Silvia Mezi, Giulia Pomati, Ilaria Grazia Zizzari, Alessandra Di Filippo, Bruna Cerbelli, Alessio Cirillo, Giulia Fiscon, Sasan Amirhassankhani, Valentino Valentini, Marco De Vincentiis, Alessandro Corsi, Cira Di Gioia, Vincenzo Tombolini, Carlo Della Rocca, Antonella Polimeni, Marianna Nuti, Paolo Marchetti, Andrea Botticelli

**Affiliations:** 1 Department of Radiological, Oncological and Pathological Science, “Sapienza” University of Rome, 00161 Rome, Italy; 2 Department of Molecular Medicine, “Sapienza” University of Rome, 00161 Rome, Italy; 3 Department of Experimental Medicine, Faculty of Medicine and Dentistry, “Sapienza” University of Rome, 00161 Rome, Italy; 4 Department of Computer, Control, and Management Engineering “Antonio Ruberti”, “Sapienza” University of Rome, 00185 Rome, Italy; 5 Department of Urology, S. Orsola-Malpighi Hospital University of Bologna, Via Palagi 9, 40138 Bologna, Italy; 6 Department of Oral and Maxillo-Facial Sciences, “Sapienza” University of Rome, 00161 Rome, Italy; 7 Department of Medico-Surgical Sciences and Biotechnology, Polo Pontino, “Sapienza” University of Rome, 04100 Latina, Italy; 8 IDI-IRCCS Istituto Dermopatico Dell’Immacolata, 00167 Rome, Italy

**Keywords:** head and neck cancer, gene mutation, immunotherapy, cytokines profile, chemokines, soluble immune checkpoints

## Abstract

Introduction: Only a minority of patients with platinum refractory head and neck squamous cell carcinoma (PR/HNSCC) gain some lasting benefit from immunotherapy. Methods: The combined role of the comprehensive genomic (through the FoundationOne Cdx test) and immune profiles of 10 PR/HNSCC patients treated with the anti-PD-1 nivolumab was evaluated. The immune profiles were studied both at baseline and at the second cycle of immunotherapy, weighing 20 circulating cytokines/chemokines, adhesion molecules, and 14 soluble immune checkpoints dosed through a multiplex assay. A connectivity map was obtained by calculating the Spearman correlation between the expression profiles of circulating molecules. Results: Early progression occurred in five patients, each of them showing TP53 alteration and three of them showing a mutation/loss/amplification of genes involved in the cyclin-dependent kinase pathway. In addition, ERB2 amplification (1 patient), BRCA1 mutation (1 patient), and NOTCH1 genes alteration (3 patients) occurred. Five patients achieved either stable disease or partial response. Four of them carried mutations in PI3K/AKT/PTEN pathways. In the only two patients, with a long response to immunotherapy, the tumor mutational burden (TMB) was high. Moreover, a distinct signature, in terms of network connectivity of the circulating soluble molecules, characterizing responder and non-responder patients, was evidenced. Moreover, a strong negative and statistically significant (*p*-value ≤ 0.05) correlation with alive status was evidenced for sE-selectin at T1. Conclusions: Our results highlighted the complexity and heterogeneity of HNSCCs, even though it was in a small cohort. Molecular and immune approaches, combined in a single profile, could represent a promising strategy, in the context of precision immunotherapy.

## 1. Introduction

Management of recurrent/metastatic head and neck carcinoma (R/M HNSCC) has been profoundly changed by the advent of immunotherapy [1,2]. The programmed death 1 (PD-1) and programmed death ligand 1 (PD-L1) axis are involved in the genesis, maintenance, and progression of HNSCC and represent the main target of immune checkpoint inhibitors (ICIs) [3,4,5]. ICIs revolutionized the standard of care in the first-line setting, as well as in platinum-refractory R/M HNSCCs [6,7,8,9,10]. Nevertheless, emerging data from clinical trials and real-world evidence showed that only a relatively small subset of HNSCC patients benefit from treatment with ICIs in the long term. In particular, patients with platinum-refractory disease are particularly difficult to treat, due to a poor response rate to immunotherapy and limited median overall survival (OS). Despite the ability of immune cells to recognize, control, and kill tumor cells, with a strong impact on the genesis, maintenance, and tumor progression, HNSCC tumor cells employ several mechanisms, in order to escape the control of the immune system [11,12]. 

The mechanisms of tumor immune resistance are complex and involve multiple factors, such as host-related ones (gender, age, distribution of body fat, gut microbiome), genetic mutations, metabolism, inflammation, and abnormal neovascularization [13]. HNSCC is distinguished by a tumor immunosuppressive microenvironment induced by a strong inflammatory component and high levels of tumor-infiltrating lymphocytes (TILs) [4,13,14,15,16], high regulatory T-cells (Tregs) [17,18], tumor-associated macrophages (TAMs), and dendritic cells (DCs) [19], with a tumor heterogeneity between different patients and an intra- and inter-tumor variability, in relation to the anatomical site of the metastasis [5]. Moreover, previous treatments, including specific oncology drugs, radical and bilateral neck dissection plus radiotherapy, and concomitant use of certain drugs, such as opioids, antibiotics, and corticosteroids, may also affect the response of HNSCC to ICIs [20,21,22].

Early detection of intrinsically resistant patients is a crucial issue in clinical practice, as it could prevent immunotherapy failure. The study of cytokines and chemokines at baseline and during immunotherapy is a repeatable and noninvasive method of monitoring the patient’s immune profile [23,24,25,26,27]. Moreover, the study of soluble factors and/or surface-bound molecules, related to the tumor microenvironment, is becoming increasingly of interest, as they are involved in the dysfunctional activity of the immune system [28]. Soluble factors are produced either by alternative splicing of mRNA, through the proteolytic shedding of extracellular regions of the cellular membrane, or released by immune cells associated with exosomes and micro-vesicles. The ability of circulating immune checkpoint molecules to regulate the immune system is manifold, either acting as a decoy for the drug directly, preventing the effectiveness of ICI antibodies, or by inhibiting the activation of infiltrating or circulating T lymphocytes by means of the PD-1/PD-L1 axis. Furthermore, soluble CD-137 negatively regulates the activation of T lymphocytes, blocking the interaction between T lymphocytes and APC [29]. Recent results suggest that the concentration of these soluble factors is lower in patients benefiting from immunotherapy, with a potential role in predicting the time to treatment failure [30,31,32]. 

The evaluation of the biomarkers of response to immunotherapy cannot fail to include the analysis of the genomic driver sensibility or primary resistance to ICIs. Increased tumor mutational burden (TMB), which is a set of non-synonymous mutations in tumors, is closely related to increased neoantigen levels, conferring the high immunogenicity and increased T-cell infiltration associated with greater sensitivity to immunotherapeutic agents, prompting the FDA to approve the anti-PD-1 pembrolizumab in pediatric and adult solid tumors with microsatellite instability status (MSI) or mismatch repair deficiency, leading to the first approval based on a specific biomarker, rather than on an organ-specific histology [33,34,35,36]. The genomic analysis of HNSCC highlighted a distinct number of mutations in genes that play key roles in cellular proliferation, differentiation, survival, and metastasis. While the functions of these genes have been studied extensively, the central role of their impact on the immune response is only beginning to be appreciated [37]. Primary HNSCC gene mutation analysis allowed for the categorization of HNSCCs into distinct groups (basal, mesenchymal, atypical, and classical), comprising cell-cycle regulating genes (CDKN2A and CCND1), genes involved in cell proliferation and survival regulation (TP53, HRAS, PIK3CA, and EGFR), genes responsible for cellular differentiation (NOTCH1), and genes involved in the modulation of adhesion and invasion of a mesenchymal-enriched subtype (Wnt signaling pathway regulator gene FAT1, protocadherin). All of these groups show statistically significant differences in recurrence-free survival. In a more recent study, HNSCCs were further categorized into five groups (non-human papilloma virus (HPV) basal, HPV classical, non-HPV classical, HPV mesenchymal, and non-HPV mesenchymal). Based on the findings, these groups were further classified into three primary subgroups: (i) the basal subgroup, characterized by an absence of an immune response related to a hypoxic tumor microenvironment; (ii) the classic subgroup, related to heavy smoking and an amplification in genetic mutations; (iii) the mesenchymal subgroup, with a high expression of mesenchymal transition markers and immune cells sharing many genetic expression features with the basal subgroup, with extensive stromal epithelial to mesenchymal transition (EMT) [38,39]. 

A combined approach, able to reflect the complexity of the relationships between the genetic profile of the HNSCC patients and their immune system, would be the most effective, rather than using a single biomarker, in order to predict response/resistance to immunotherapy. Genomic profiling of HNSCC cancers may enhance the predictive utility of the TMB profile and allow evaluation of how the patterns of gene expression regulate the immunophenotype, thus affecting the immune response of R/M HNSCCs and facilitating the establishment of a personalized combination of immunotherapy approach in genomically-defined subgroups. 

To this purpose, we conducted an exploratory combined evaluation, aiming to identify the implications of genetic alterations and their relationship with the basal and dynamic changes of the immune profile and with the response to immunotherapy treatments and clinical outcomes. A large spectrum of circulating molecules, including cytokines, chemokines, soluble immune checkpoints, molecules of adhesion, and indoleamine-2,3-dioxygenase (IDO, an important microenvironmental factor suppressing antitumor immune responses), were analyzed through a network analysis, in the serum of patients with R/M HNSCC, before and during ICIs treatment [40]. These were then correlated with the analysis of genetic alterations of the patients. The primary objective was to identify any possible biomarker that could influence outcomes in the responders and refractory patients affected by HPV-negative platinum-refractory HNSCC treated with immunotherapy.

## 2. Materials and Methods

### 2.1. Patient Enrolment 

Patients with R/M platinum-refractory HPV-negative HNSCC, eligible for immunotherapy (regardless of PDL1 expression), who started treatment with the anti-PD1 nivolumab were enrolled. Data regarding age, gender, baseline Eastern Cooperative Oncology Group performance status (ECOG PS) evaluated before the start of nivolumab, history of tobacco smoking and alcohol abuse, tumor site(s), previous locoregional treatment, previous first-line chemotherapy, and histology were collected. Contrast-enhanced computed tomography (CT) scan and contrast-enhanced magnetic resonance imaging (MRI) and/or CT-PET (positron emission tomography), when appropriate, were used to stage patients. Inclusion criteria were: age 18 years or older, histologically confirmed R/M HNSCC, localization of the primary tumor in the oral cavity, oropharynx, and larynx. The disease had to be non-susceptible to other local therapies with curative intent (surgery and/or radiotherapy) and had to have progressed on or within 6 months from the last platinum dose administered in a first-line setting. Patients fit for immunotherapy with adequate bone marrow, liver, and renal function, ECOG PS ≤ 2, were included in the study. All patients provided an informed consent to be included in the study and for blood samples to be collected. Patients who received anti-neoplastic immunotherapy for other previous or concomitant pathologies, with PS > 2, with a non-squamous histology, with uncontrolled autoimmune or infectious diseases, or not compliant with protocol requirements were excluded from the study. Protocol approval from the local ethics committee was obtained (CE 4421). Nivolumab was administered at a dose of 240 mg every 15 days, until either disease progression or unacceptable toxicity was recorded. Radiological response was assessed according to immune RECIST criteria every three months. Early progression was defined as the occurrence of disease progression within 3 months from the start of immunotherapy. Progression-free survival (PFS) was defined as the time, in months, from the start of immunotherapy until the occurrence of either progression or death, or the date of the last follow-up. OS was the time, in months, from the start of immunotherapy to the date of death or of the last follow-up visit. Based on the response to immunotherapy, patients were classified as non-responders if early progression occurred or responders if the response was at least that of stable disease (SD) for at least 4 months.

### 2.2. Samples Collection 

Peripheral blood samples were collected from 10 R/M HNSCC patients, both at baseline (T0) before the first administration of nivolumab and after the second cycle of the anti-PD1 therapy (T1). At the same time, after centrifugation, serum samples were collected and stored at −80 °C until use. 

### 2.3. Circulating Soluble Molecules 

For each patient, 50 µL of serum was used and added to a 96-well plate, together with a mixture of magnetic beads coated with antibody, according to the manufacturer’s instructions. Serum concentration of cytokines (TNF alpha, IFN alpha, IFN gamma, IL1 alpha, IL1 beta, IL10, IL12p70, IL13, IL17A, IL4, IL6, GM-CSF), chemokines (MCP1, MIP-1alpha, MIP-1 beta, IL8, IP10), soluble immune checkpoints (BTLA, sCD137 sCD27, sCD28, sCD80, sCTLA-4, sGITR, HEVM, sLAG3, sPD1, sPDL-1, sPDL-2, sTIM3), adhesion molecules (sE-selectin, sP-selectin, sI-CAM-1), and IDO were evaluated. The concentration of the molecules was dosed through a multiplex assay using the Human Immuno-Oncology Checkpoint 14-plex ProcartaPlex panel 1 (catalog number: EPX14A-15803-901) (eBioscience) (Thermo Fischer Scientific, Waltham, MA, USA) and the Human Immuno-Oncology Checkpoint 14-plex ProcartaPlex Human Inflammation panel (catalog number: EPX200-12185-901). Samples were analyzed using Luminex 200 platform (BioPlex, Bio-Rad, Hercules, CA, USA). Data (expressed in pg/mL) were analyzed with Bio-Plex Manager Software. 

### 2.4. Statistical Analysis 

A total of 34 molecules from 10 patients (of which, 5 were classified as responders to the therapy, with 5 as non-responders to the therapy and 3 classified as alive versus 7 classified as dead) were analyzed. Data were first pre-processed through the application of a logarithmic transformation, and experimental differences between the expression levels in the responder patients and non-responder patients were then tested for statistical significance via Mann–Whitney test at two different times: T0 (i.e., basal) and T1 (i.e., after three months). Statistical significance was defined by a *p*-value ≤ 0.05. Given the exploratory nature of the study, corrections for multiple testing were not applied.

### 2.5. Connectivity Analysis 

In order to investigate the relationships between soluble immune-related molecules levels and therapy response, a connectivity map was first obtained by calculating Spearman correlation coefficients between the expression profiles of the investigated circulating molecules available from 10 patients and the distribution of their corresponding therapy response values. In order to further investigate the differences, in terms of the connectivity of the analyzed molecules in responder and non-responder patients, two connectivity matrices were built by calculating the Spearman correlation coefficients among each pair of molecules, one for responder patients and one for non-responder ones, and were rendered as two connectivity maps where correlation values increased from red to blue. Correlation values with *p* ≤ 0.05 were considered to be statistically significant. The two corresponding networks of connectivity were then constructed, in which nodes represented molecules, and a link occurred between them if the absolute value of Spearman correlation between their expression levels was greater than the selected threshold (i.e., the 85th percentile of the overall distribution corresponding to 0.8) and statistically significant (*p*-value ≤ 0.05). All the connectivity networks, along with their corresponding values of correlation and statistical *p*-values, were detailed as edge lists in Supplementary Table S1 (for time T0) and Supplementary Table S2 (for time T1). A further exploratory data analysis of the 34 molecule expression levels from 10 patients grouped by patient status (alive versus dead) was performed. To investigate the difference in the patterns of molecule connectivity, even in terms of patient status, two connectivity maps between each pair of molecule expression values were built, one for alive patients and one for dead patients, both at T0 and at T1, respectively.

### 2.6. Comprehensive Genomic Cancer Profiling 

Genomic cancer profile was defined through Foundation One^®^ CDx, a next generation sequencing tissue-certified comprehensive genomic profiling service for all types of solid tumors, capable of detecting 4 main classes of genomic alterations (substitution of bases, insertions and deletions, alterations in the number of copies, gene rearrangements) in 324 tumor-related genes, as well as genomic signatures, including TMB and MSI [41,42,43,44]. Formalin-fixed, paraffin-embedded samples of the tumor were collected at the first diagnosis for each patient and were used for genomic cancer profiling.

## 3. Results

A total of 10 consecutive patients with HPV-negative R/M platinum refractory HNSCC who started nivolumab between December 2018 and July 2019 were included. All of the patients received platinum-based chemotherapy in a first-line setting and underwent disease progression at or within 6 months from the last platinum dose. Their clinical and pathological features are reported in Table 1. Median age was 66 years (range, 46–66 years), and eight patients were male. Baseline ECOG PS was 0 and 1 in two and eight patients, respectively. The primary tumor site was the oropharynx in one patient, the larynx in five patients, and the oral cavity in four patients. Grading was G2 and G3 in four and six patients, respectively. HPV status was negative in all of the patients, including the one with oropharyngeal cancer. One patient had exclusively recurrent disease not curable with locoregional treatments, while the other patients had at least one metastatic site.

### 3.1. Outcomes

The median follow-up time was 27 months (22–31). The median PFS was 3 (1–33) months and median OS 6.5 (2–33) months. Five (50%) patients were classified as non-responders, as early progression occurred (cases 1–5) (Table 2). Among the other cases, three patients underwent progression within 6 months of starting nivolumab (cases 6, 7, and 10), while the remaining two patients obtained a prolonged response (20–33 months). Case number 8 was still undergoing nivolumab treatment at the time of writing the present manuscript. Four patients received an additional line of chemotherapy after nivolumab progression (cases nos. 2, 4, 7, and 9). Three patients (cases 2–8–9) were alive at the time of the last visit, while the other seven patients succumbed to the disease (Table 2).

### 3.2. Early Progressive Disease (Cases 1–5) 

The median PFS was slightly low (2 months), and the median OS was 11 months (3–21). Case 2 showed a prolonged response to a third-line treatment, based on weekly paclitaxel, which resulted in a long survival (Table 2). In each patient who experienced early progression, both TMB and MSI were defined (Table 3): MSI was stable in all of the patients, and TMB was low in four out of five patients, with six mutations or less reported. In the last patient (case 1), the TMB was intermediate (eight mutations/Mb). Alteration in TP53 was evidenced in all the patients with early progression. Alterations in other tumor suppressor genes (ARID1A and NOTCH genes) were reported. Alteration in NOTCH genes, involved in cellular differentiation, occurred in three out of five patients, showing early progression. Mutation in genes involved in genomic stability maintenance, always in association with other mutations, were also recorded in two patients. One of them showed the BRCA1 mutation (case 1). In another patient who experienced early progression, TERC amplification was recorded (case 3). Early progression occurred in one patient with ERB2 amplification (case 3). Other genes of the EGFR family (PI3K amplification, SOX2 amplification) were also amplified in the same case. Three out of five patients (cases 1, 3, and 5) showed mutation/loss/amplification of genes involved in the cyclin-dependent kinase (CDK) pathway. Alteration in one gene involved in cell adhesion, cytoskeletal organization, migration, and angiogenesis was evidenced (EPHB4 amplification). In case 1, the simultaneous alteration of the genes involved in the chromatin remodeling, genomic stability, cell-cycle control, and the alteration of the antiapoptotic BCL2L1 and NFE2L2 genes, involved in the response to oxidative and electrophilic stress, was evidenced. The genomic analysis reported six mutations in ARID1A, BCL2L1, BRCA1, CDKN2A/B, NFE2L2, and Tp53 (case 1). The genomic analysis of case 2 detected two mutations: NOTCH1 and Tp53. In case 4, the same mutation in Tp53, plus the RB1 and NOTCH2 mutations, were detected. The genomic analysis of case 3 detected 13 mutations (the greatest number of mutations in our series) involving TP53, the NOTCH pathway, and the EGFR pathway, genes involved in the dysregulation of cell-cycle control and cell adhesion. This is the only case with mutations in SOX2 and ERB2. The genomic analysis of case 5 detected five mutations: CDKN2A/B, SMAD4, which are involved in cell-cycle control, TERT (involved in genomic stability), NOTCH1, and TP53 (Table 3). 

### 3.3. Responders Patients (Cases 6–10) 

In patients who achieved a response to treatment, the median PFS was 13.6 months (4–33), and the median OS was 16.4 months (5–33). MSI was stable in all of the patients; however, TMB was high in two patients (cases 8 and 9). Patient no. 9 was a long responder with a PFS of 20 months and was alive at the time of writing (OS 39 months) (TMB 15 muts/MB). Alteration in TP53 and in several genes involved in the promotion of invasion were evidenced. Moreover, loss of PTEN was reported. Patient no. 8 achieved a considerable long-lasting response, with a TMB of 30 mut/MB. The ICI treatment was still ongoing, and the PFS was 33 months. In this case, no TP53 alterations were evidenced, while dysregulation in cell-cycle control via cyclin-dependent kinase-6 and SMAD4 (a signal transduction protein altered in response to TGF beta signaling) were reported, as well as PI3K alteration. Moreover, alteration in SPEN (a repressor of NOTCH pathway) was evidenced. Four out of five responder patients showed mutations in the genes involved in PI3K/AKT/PTEN pathways, and three of them achieved a partial response to nivolumab, as recorded at the first instrumental evaluation (cases 6 and 8–10). All patients showed a dysregulation of the CDK-cyclin pathway associated with the loss of cell-cycle control. The genomic analysis detected 9 mutations in patient no. 6, including SOX2. In the case of patient no. 10, the genomic analysis detected four mutations, CDK2A/B and MTAP, involved in proliferation of cancer cells, PBRM1, which is a tumor suppressor reported to predict response in urothelial carcinoma, and PIK3C2G. 

### 3.4. Statistical and Connectivity Analysis of Circulating Molecules 

**Figure 1:**
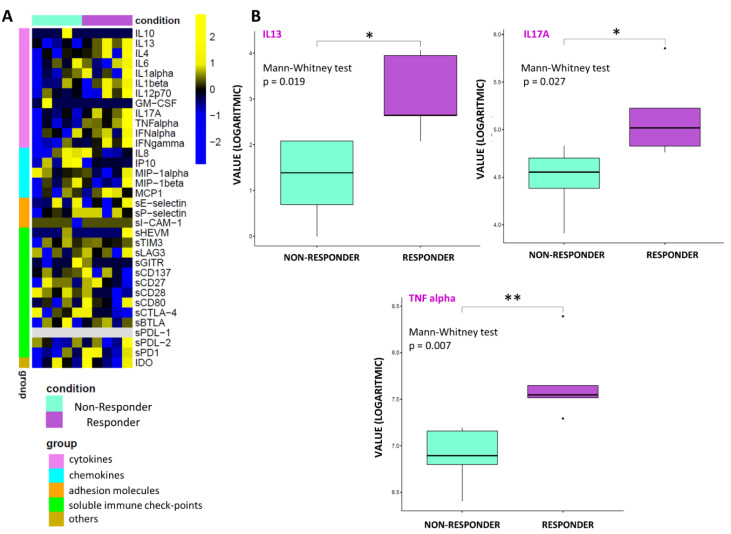
Statistical analysis at T0. (**A**) Heatmap of molecule expression levels (logarithmic scale) at T0 across 10 patients, grouped by therapy responder (violet bars) and non-responder (water blue bars). Colors represent different expression levels, increasing from blue to yellow. (**B**) Boxplot of molecule expression levels (logarithmic scale) in 5 responder patients (violet box) and 5 non-responder ones (water blue box) at T0. *p*-values (*p*) were obtained by performing a Mann–Whitney test for unpaired samples. Only molecules showing a statistically significant difference between the two groups are shown. Legend: * *p* ≤ 0.05, ** *p* ≤ 0.01.

**Figure 2:**
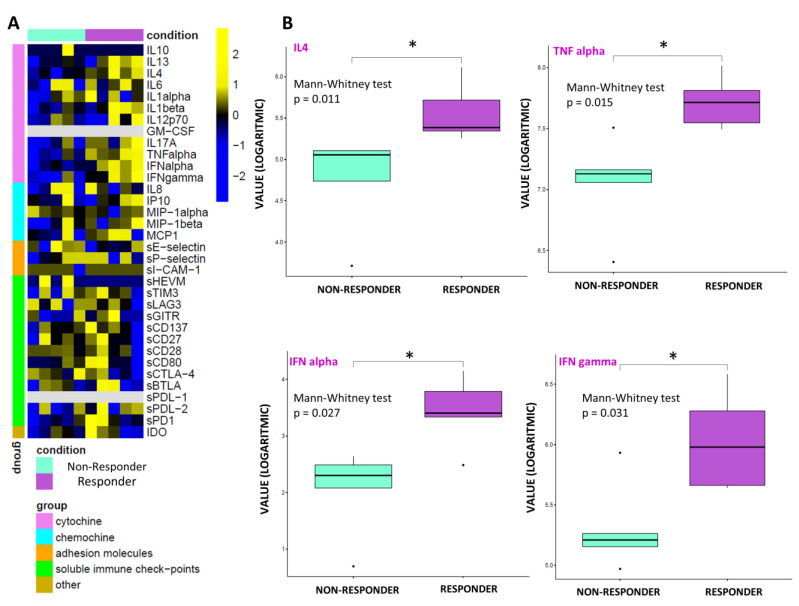
Statistical analysis at T1. (**A**) Heatmap of molecule expression levels (logarithmic scale) at T1 across 10 patients, grouped by therapy responder (violet bars) and non-responder (water blue bars). Colors represent different expression levels, increasing from blue to yellow. (**B**) Boxplot of molecule expression levels (logarithmic scale) in 5 responder patients (violet box) and 5 non-responder ones (water blue box) at T1. *p*-values (*p*) were obtained by performing a Mann–Whitney test for unpaired samples. Only molecules showing a statistically significant difference between the two groups are shown. Legend: * *p* ≤ 0.05.

By performing an exploratory data analysis of the 34 molecule expression levels from 10 patients, grouped by therapy response, no clear separation, in terms of the overall molecule expression levels across to the two classes, was detected, both at T0 (Figure 1A) and T1 (Figure 2A). Yet, we observed a statistically significant over-expression in the responder patients of the cytokines IL13, IL17A, and TNF alpha at T0 (Figure 1B) and over-expression of IL4, TNF alpha, IFN alpha, and IFN gamma at T1 (Figure 2B). 

**Figure 3:**
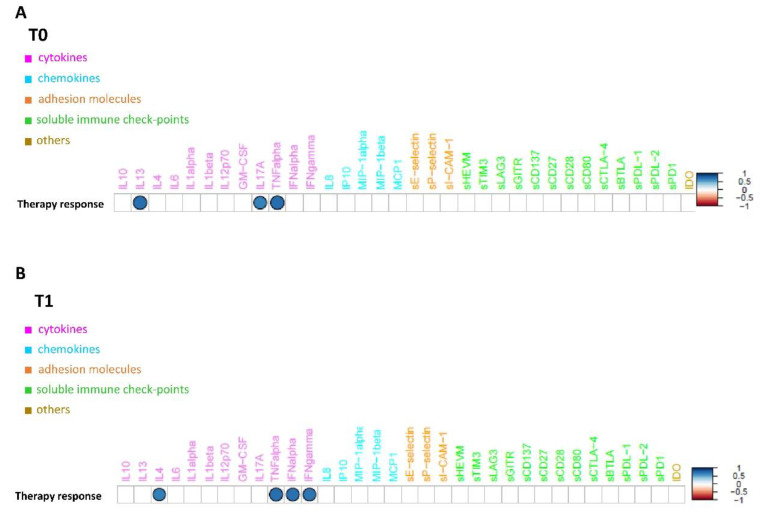
Connectivity map between molecules’ profiles and therapy response values at T0 (**A**) and at T1 (**B**). Statistically significant Spearman correlations (*p*-value ≤0.05) are reported. In the plot, circles are scaled and colored according to the correlation values, increasing from red (negative correlation) to blue (positive correlation). Molecules are grouped and ordered according to the functional group reported in the legend.

These results are confirmed by the correlation analysis computed between the expression profiles of the under-study circulating molecules available from 10 patients and the distribution of their corresponding therapy response values, which unveiled a strong positive and statistically significant (*p*-value ≤ 0.05) correlation between the group of above-mentioned cytokines and the therapy response both at T0 (Figure 3A) and at T1 (Figure 3B). Conversely, no clear separation, in terms of overall molecule expression levels across the two classes, alive vs dead, was detected, both at T0 (Supplementary Figure S1A) and T1 (Supplementary Figure S1B). The correlation analysis computed between the expression profiles of the under-study circulating molecules and the distribution of their corresponding patient status at T1 unveiled that the only molecule with a strong negative and statistically significant (*p*-value ≤ 0.05) correlation with alive status was sE-selectin (Figure 2A). This appears to be in accordance with its down-regulation in patients who were alive at T1 with an almost statistically significant *p*-value (=0.06) (Figure 2B).

### 3.5. Connectivity Network Analysis between Circulating Molecules in Responder and Nonresponder Patients 

**Figure 4:**
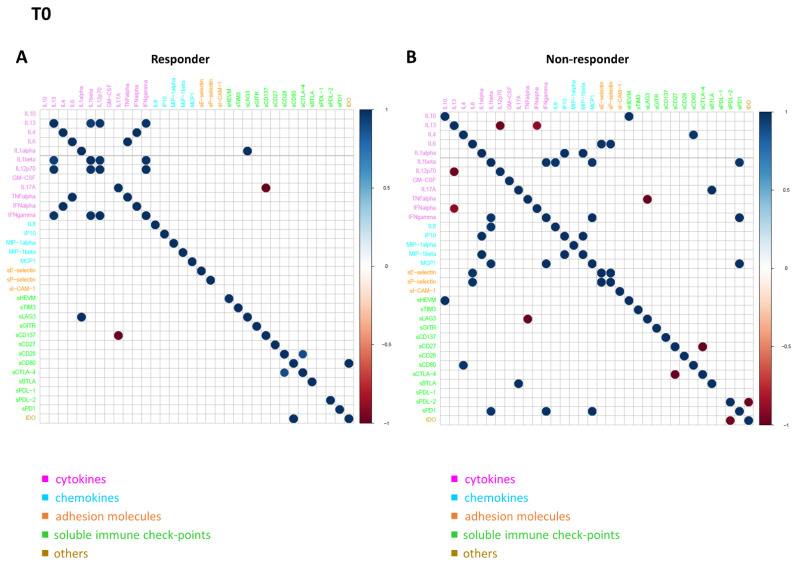
Connectivity map between molecules in responder (**A**) and non-responder (**B**) patients at T0. Statistically significant Spearman correlations (*p*-value ≤ 0.05) are reported. In the plot, circles are scaled and colored according to the correlation values, increasing from red (negative correlation) to blue (positive correlation). Molecules are grouped and ordered according to the functional group reported in the legend.

**Figure 5:**
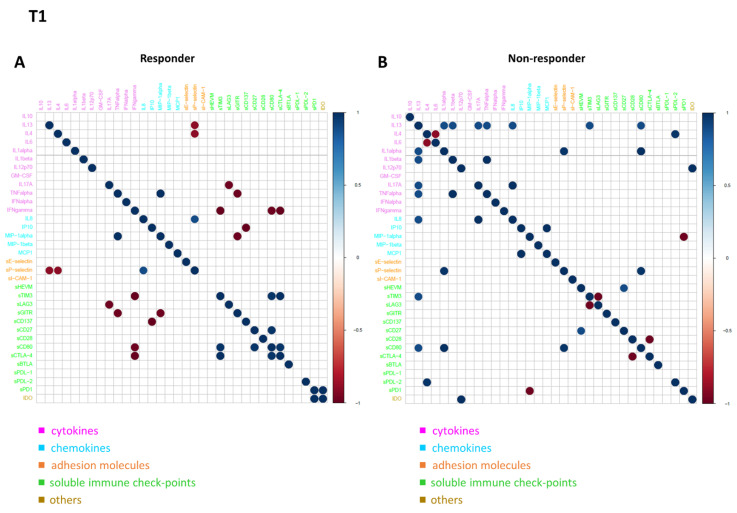
Connectivity map between molecules in responder (**A**) and non-responder (**B**) patients at T1. Statistically significant Spearman correlations (*p*-value ≤ 0.05) are reported. In the plot, circles are scaled and colored according to the correlation values, increasing from red (negative correlation) to blue (positive correlation). Molecules are grouped and ordered according to the functional group reported in the legend.

In order to further investigate the difference in the patterns of molecule connectivity, in terms of therapy response, two connectivity maps between each pair of molecule expression values were built, one in the responder patients and one for non-responder patients, both at T0 (Figure 4A,B) and at T1 (Figure 5A,B), respectively. From these maps, different connectivity patterns were observed, both at T0 and T1. At T0, moving from the non-responder to the responder group, there was an intensification in most of the cytokine correlations and, in some cases, an inversion of the correlation sign, from negative to positive (e.g., IL13 in responder patients appears to be strongly positive correlated with IL12p70, while in non-responder patients, this correlation is negative, meaning that if IL13 is high the other one is low and vice versa). In regards to T1, moving from non-responder to responder patients, a lack of connectivity was observed in most of the cytokines (e.g., the connections of the pro-inflammatory cytokines IL13, IL1 alpha, and IL1 beta; most of the connections of the other groups of molecules, including sPD1 and sCD80). 

**Figure 6:**
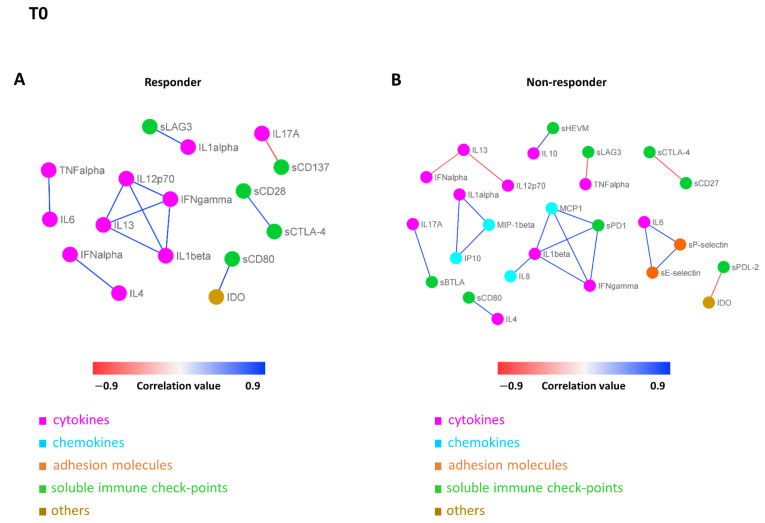
Connectivity network between molecules in responder (**A**) and non-responder (**B**) patients at T0. In each network, nodes represent molecule, and a link occurs between two nodes if the absolute value of Spearman correlation between their expression levels is statistically significant (*p*-value ≤ 0.05) and greater than a selected threshold (i.e., the 85th percentile of the overall distribution corresponding to 0.8). Nodes are colored according to the functional groups reported in the legend, whereas edge colour indicates positive (blue) or negative (red) correlation values.

**Figure 7:**
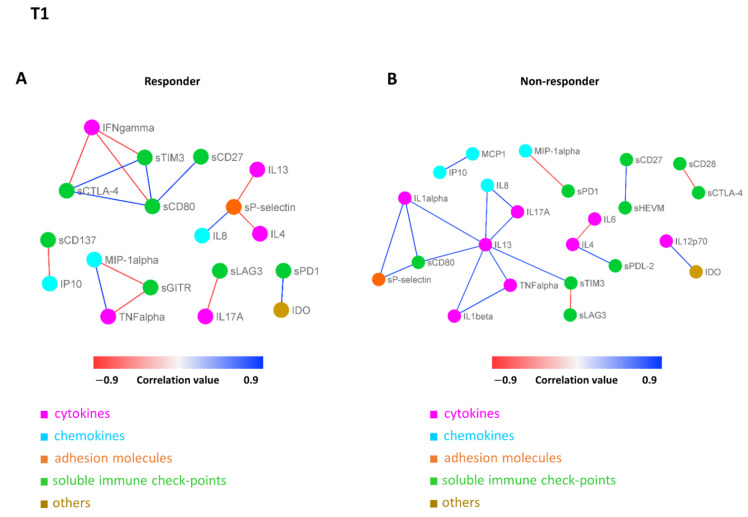
Connectivity network between molecules in responder (**A**) and non-responder (**B**) patients at T1. In each network, nodes represent molecule, and a link occurs between two nodes if the absolute value of Spearman correlation between their expression levels is statistically significant (*p*-value ≤ 0.05) and greater than a selected threshold (i.e., the 85th percentile of the overall distribution corresponding to 0.8). Nodes are colored according to the functional groups reported in the legend, whereas edge colour indicates positive (blue) or negative (red) correlation values.

These differences were more evident when the connectivity maps for responder and non-responder patients were rendered as two corresponding connectivity networks (Figure 6. for T0, Figure 7 for T1), in which two nodes are connected if their expression profiles are statistically significant (*p* ≤ 0.05) and exceed (as an absolute value) a selected correlation threshold (i.e., the 85th percentile of the overall distribution corresponding to 0.8). Looking at the network of connectivity at time T0, a total of 12 statistically significant connections (correlations) were identified in responder patients (Figure 6A and Supplementary Table S1, first sheet), 21 connections in non-responder patients (Figure 6B and Supplementary Table S1, second sheet), and only 2 common connections to both networks (Supplementary Table S1, third sheet). 

In regards to T1, we identified a total of 16 statistically significant connections in responder patients (Figure 7A and Supplementary Table S2, first sheet), 20 connections in non-responder patients (Figure 7B and Supplementary Table S2, second sheet), and no common connections between them. These findings demonstrated a distinct signature, in terms of network connectivity of the molecules characterizing responder patients, as opposed to non-responder ones.

The connectivity maps between each pair of molecule expression values, one for alive patients and one for dead patients, both at T0 (Supplementary Figure S3) and at T1 (Supplementary Figure S4), respectively, evidenced a different connectivity between alive and dead patient groups with no common connection, both at T0 and T1. In both cases, moving from the connectivity map of the dead patient group to the alive one, a lack of connectivity was observed in most of the cytokines and immune checkpoints, as well as the appearance of new negative correlation values among some soluble immune checkpoints, such as between GM-CSF and sGITR at T0 (Supplementary Figure S3A) or between sPD1 and sCD28, sHEVM, and sCD80 at T1 (Supplementary Figure S4A). 

The resulting networks from the connectivity analysis with the corresponding values of correlation from both patient groups at T0 and at T1 were reported in Supplementary Table S3.

## 4. Discussion

Considering the complexity of R/M HNSCCs, a combined profile, including genomic and immune evaluations, could be a promising approach to evaluate the mechanisms driving resistance to immunotherapy and to tailor individual treatments. In this series, the R/M HNSCCs analyzed had a heterogeneous immune and genomic profile. This heterogeneity explains how complex it is to identify new robust predictive biomarkers of both resistance and response to immunotherapy. While it is necessary to broaden the research on a larger number of patients, this small series still provides several important insights that deserve further investigation. The group of patients analyzed was homogeneous, in terms of prognosis and previous treatments administered, and survival data are mature. Platinum-refractory HPV-negative HNSCC is confirmed as a poor prognosis disease, burdened by multiple mechanisms of resistance, although they are not fully understood and poorly predictable in each individual patient, for which the treatment options are still limited. Overall, a low response rate to ICI treatment was reported, and a high percentage of cases was burdened by early progression and a poor mPFS and OS. Nevertheless, the duration of the response was variable, and two patients achieved a prolonged response to treatment with ICI, while another patient was still on treatment after 33 months. This variability in outcomes confirms the assumption that similar prognostic groups from a histological and clinical point of view underly relevant biological differences, which significantly affect prognosis and response to treatment. The biological differences may be related to several factors, including the inter-individual variability in the immune system, the genomic driver primary resistance, and the relation to acquired mechanisms of resistance to immunotherapy. Inter-individual variability and fitness of the immunological framework could influence the individual response to immunotherapy treatment and the onset of autoimmune and inflammatory disorders. Host (e.g., sex and age) and environmental factors (e.g., smoking and medical history including asymptomatic cytomegalovirus infection, past infections, vaccinations, colonization by intestinal and nasal bacteria) shape the immune system, making individuals more or less prone to diseases and equipped to deal with pathogens and neoplastic cells, thus affecting the course of diseases and their outcomes [45,46]. In addition, genetic factors also play a key role in regulating and individualizing the immune system by changing the expression of key immune response molecules. 

### 4.1. Soluble Immune Profile

In this series, the sIC profile was heterogeneous and varied widely between patients with early progression and responders and based on clinical outcome. Heatmap analysis of molecule expression levels evidenced, in responder patients, an increase in chemokine and interleukin levels associated with a lower level of immune checkpoint and adhesion molecules versus resistant ones, both at baseline and at T1. Among the large spectrum of molecules analyzed, a strong, positive, significant correlation between basal overexpression of IL13, IL17A, and TNF alpha and the immunotherapy response between the two groups was shown at T0 (Table 4, Figure 3A) [47,48,49,50,51,52,53]. 

IL13 facilitates the recruitment of leukocytes, particularly neutrophil granulocytes and monocytes, stimulating the expression during the inflammation of adhesion proteins and chemokines on the endothelium and is involved in the process of fibrosis by stimulating macrophages and fibroblasts to produce collagen. In macrophages, it also stimulates the production of TGF-β [47]. IL-17A, produced mainly by T helper 17 cells, is involved in host defense against microbial organisms and in the development of immune-mediated inflammatory diseases and cancer [48]. Tumor necrosis factor (TNF)-alfa is involved in the acute phase of systemic inflammation and is a key regulator of the immune system, as well the cellular antiapoptotic response. Therefore, our data underline that an essential prerequisite for the response to immunotherapy treatment was a readily activable immune system on both innate and adaptive immunity, “inflammable” with acute phase protein expression [49]. Moreover, it was observed that patients who obtained disease control had a strong, significant, positive overexpression of IL4, TNF alpha, IFN alpha, and IFN gamma at T1 (Table 4, Figure 3B) [47,48,49,50,51,52,53]. The interleukin IL4, produced mainly by T-cells, induces B-cell proliferation, expands selected B-cell subgroups, and causes Th2 differentiation and proliferation [50,51]. In this scenario, an intriguing role could be played by tertiary lymphoid structures (TLSs). TLSs reflect the lymphoid neogenesis of organized cellular aggregates resembling secondary lymphoid organs (SLO), which occur in peripheral nonlymphoid tissues, following long- lasting exposure to inflammatory signals mediated by chemokines (i.e., CXCl13) and cytokines (i.e., Il7), generating a specific immune reaction outside SLO at the tumor site [54]. TLSs facilitate the influx of immune cells into the tumor site and have, therefore, elicited interest as a means of improving anti-cancer immunity, showing a favorable treatment response in patients treated with immunotherapy. Moreover, TLSs correlates with disease evolution and with favorable clinical outcome, showing paramount importance as a prognostic factor [55]. Lastly, the ability to induce TLS formation through various pharmacological approaches represents a way to increase the sensitivity of immune cold tumors to immunotherapy [56]. Our data show a strong and maintained immunomodulatory stimulus of humoral immunity and adaptive T-cell response involving: (i) the growth and active proliferation of B- and T-cells, in particular, Th2, macrophages, and natural-killer; (ii) the regulation of class II MHC production associated with the enhancement of the expression of class I and II glycoproteins of the major histocompatibility complex; (iii) the expression of some adhesion molecules on endothelial cells, in particular, VCAM-1, with the consequent increase of links between lymphocytes, monocytes, and eosinophils representing the essential requirement for control cancer progression by maintaining the response to immunotherapy treatment. In agreement with the literature, statistical analysis evidenced a trend in lower levels in sICs and adhesion molecules in responders, confirming their key role in immune system dysfunction. Leung et al. demonstrated a direct correlation between serum levels of soluble CTLA-4 and outcomes in patients with metastatic melanoma under treatment with ipilimumab [57]. In a similar fashion, Zizzari et al. observed that low levels of sPDL-1, sPDL-2, sTim3, sCD137, and sBTLA were correlated with a longer response to anti-PD1 treatment in NSCLC patients [30]. Furthermore, an exploratory study showed a correlation between sPDL-1 and sCTLA-4 and poor response to tyrosine kinase inhibitor (TKI) treatment in metastatic renal cancer [58]. In a study involving a large population of metastatic renal cancer, sPDL-2 was the strongest predictor of recurrence, whereas high sBTLA and sTIM3 were associated with decreased survival [32]. Moreover, the significant increase of the cell adhesion molecule sE-selectin levels in the dead patients group makes it a candidate as a marker of increasing metastatic potential and a worse prognosis. 

The immune system is composed and regulated by numerous interconnected molecules. Each individual molecule has multiple molecules with which it can interact, making a bidirectional signaling possible in the network; depending on the context, a single molecule could, therefore, be either pro-inflammatory or anti-inflammatory. So, it seems unjustified to analyze the role of a single molecule in a network, even though the huge amount of available data could not easily be interpreted by means of classical statistical methods. In this scenario, a novel approach, such as network analysis, could help to understand the multitude of interactions between the different circulating molecules, in order to define some peculiar profile in responder and non-responder patients. Through connectivity network analyses, different connectivity patterns were observed, both at T0 and T1, based on response to immunotherapy. In responder patients, in contrast to non-responder ones, there is an intensification of most of the cytokine correlations and, in some cases, an inversion of the correlation sign, from negative to positive. Of considerable interest is the fact that only two common connections (correlations) to both networks were evidenced at T0, and no common connection emerged between the responder and non-responder patients at T1. All of these findings pointed to a distinct signature, in terms of the network connectivity of the circulating soluble adhesion molecules, soluble immune checkpoints, cytokines, and chemokines in responder patients, in opposition to non-responder ones, as well in both the alive and dead patient groups. These peculiar signatures seem to represent a promising tool to identify responders and/or resistant patients, although these data will need to be validated on a greater number of patients. 

### 4.2. Genomic Profile

The relation between the genomic signature and response to immunotherapy has already been extensively studied in several solid tumors [33,34,59]. The accumulation of genetic and epigenetic signaling pathway alterations in genes, causing the acquisition of different cancer phenotypes, highlighted a distinct number of oncogenes targeted by mutations, leading to the activation of tumor suppressor pathways, including p53, Rb/INK4/ARF, and Notch in HNSCCs [60]. This small group confirmed the central role of TMB in predicting increased sensitivity to immunotherapeutic agents. High values of TMB (>20 muts/MB) occur in 5.8% of HNSCC patients, with no significant difference between HPV-positive and HPV-negative disease [59,61,62]. TMB was elevated in 2/10 HPV-negative patients of this series; both of these patients showed a long-lasting response to nivolumab. These data are in agreement with a large-scale retrospective analysis of immune checkpoint inhibitor efficacy in HNSCCs, which reported significantly improved OS and PFS in patients with tumors harboring TMB ≥13 Muts/Mb, compared to those with tumors with TMB 10 Muts/Mb, who have been reported to experience significantly longer OS, compared to those with tumors with TMB < 13 Muts/Mb15 [63]. In addition, patients with EBV/HPV-negative tumors with TMB >10 Muts/Mb have been reported to experience significantly longer OS, compared to those with tumors with TMB <5 Muts/Mb (20.0 vs. 6.0 months, *p* = 0.01 (*n* = 81)) [24]. Moreover, TMB tends to be higher in smokers, which characterizes the large prevalence in our patients, compared to non-smokers (11.5 vs. 5.6 muts/Mb, respectively) [58,64]. 

The genomic profile of HNSCC patients was extremely variable. Multiple mutations were detected as affecting the genes involved in major signaling pathways, resulting in the alteration of multiple intracellular pathways, which could have a different weight on tumor progression and response to ICIs (Table 5). 

From the TCGA data, the two most predominant gene alterations and mutations reported were TP53 and CDKN2A, which were largely absent in HPV positive tumors. However, both HPV-positive and HPV-negative HNSCC tumors shared similar amplifications in PIK3CA and SOX2. In agreement with this, the most frequent genomic alteration occurred in TP53 (8/10). About 40–50% of human cancers carry deleterious mutations in p53 [65]. TP53 mutations have been reported in 38–58% of HNSCCs [66,67]. An association between TP53 mutations and shorter recurrence-free survival in patients with HNSCC has been reported, while another study found that truncating TP53 mutations, but not missense mutations, were associated with worse OS and PFS, when compared to wild-type TP53 [66,67,68]. On the other hand, TP53 mutations were associated with worse outcomes in patients treated with immunotherapy [69]. Loss or mutation of p53 can affect the recruitment and activity of myeloid and T-cells, allowing for immune evasion and promoting cancer progression. p53 can also have an altered function in immune cells, resulting in various outcomes that could either impede or support tumor development. Perturbations in p53 contribute to the ability of tumor cells to escape from immune surveillance, thus promoting an immunosuppressive environment [70]. The vital role of Rb pathway is evidenced by the inactivation of CDKN2A, encoding the cell-cycle modulators p14/Arf/INK4B and p16/INK4A in a large proportion of head and neck malignancies. CDKN2B encodes for the tumor suppressor p15INK4b. Both p15INK4b and p16INK4a bind to and inhibit CDK4 and CDK6, maintaining the growth-suppressive activity of the Rb tumor suppressor; loss or inactivation of either p15INK4b or p16INK4a contributes to the dysregulation of the CDK4/6-cyclin-Rb pathway and to the loss of cell-cycle control [71,72]. CDKN2A/B alterations occur in 17–57% of human HPV-negative HNSCCs [62,64,73,74]. Cyclin-dependent kinase (CDK) pathway dysregulation was frequent in this study population (8/10). Three out of the five early progressing patients showed a mutation/loss/amplification of the genes involved in CDK signaling. Concurrent deletion of CDKN2A and CDKN2B has been reported in 30% of HPV-negative cases in HNSCC [62]. In contrast, CDKN2A genomic alterations have not been observed in HNSCCs with positive HPV status [62,64,73,74].

This study showed that alterations in the NOTCH1 and NOTCH2 genes occurred in 3/10 patients with primary resistance to treatment. Other studies reported NOTCH1 mutations in 15–26% of HNSCCs, suggesting that NOTCH1 acts as a tumor suppressor in HNSCC [75,76]. Thus, the NOTCH pathway could play an important role in HNSCC development. NOTCH1 likely plays a bimodal role in HNSCCs, with inactivating mutations indicating a tumor suppressor role and activating mutations and upregulation consistent with an oncogenic role. The NOTCH receptor, which is expressed on tumor cells, binds to the NOTCH ligand on myeloid-derived suppressor cells (MDSCs), resulting in the improvement of the cancer stem cell capacity and the immune-escape mechanism. Moreover, NOTCH signaling is involved in T-cell-mediated anti-tumor immunity [77]. Tumor cells are often able to suppress NOTCH signaling, in order to evade T-cell-mediated killing. In contrast to our observations, NOTCH mutation has recently been evaluated as a possible predictive biomarker of the clinical benefit of immunotherapy in NSCLC [78]. Somatic frame shift mutations of common tumor suppressor genes (NOTCH1 and SMARC4) were also observed in HPV-negative patients responsive to anti-PD-1/PD-L1 agents [79]. NOTCH is also related to EMT, which has been linked to therapeutic resistance, invasion, and metastasis [80,81,82,83]. 

Alterations in PI3K pathway were quite frequent in this series (5/10 patients), with a high frequency observed in responders. In one study, PIK3CA alteration was associated with lymph node metastasis, and PIK3CA mRNA was associated with the tumor stage [84,85]. PIK3CA amplification was found to be linked with cancer relapse in patients with HNSCC, without nodal involvement [85]. Moreover, in a recent study, PI3KCA alterations were correlated to good OS, representing a favorable predictive factor of immunotherapy efficacy in HNSCC patients, in agreement with our observations [69]. 

Notably, one case was characterized by early progression harboring BRCA1 mutation. BRCA1 and BRCA2 are tumor suppressor genes with a key role in DNA repair processes and cell-cycle checkpoints, in response to DNA damage, whose mutations may induce genomic instability, cell-cycle dysregulation, and accumulation of other mutations, thus increasing the risk of cancer development [86,87,88,89]. In the same patient, ARID1A mutations occurred. In human cancers, ARID1A drives cancer development and defines IFN responsiveness and immune evasion, resulting in poor immunotherapy responses [90]. 

SOX2 alteration was reported in 2/10 patients, regardless of response to treatment. SOX2 was recently reported to play a key role in HNSCC immune evasion. In an “in vivo study”, SOX2 expression in HNSCC tumor cells led to a decrease in CD8+ T-lymphocyte infiltration and promoted tumor growth through the suppression of stimulation of interferon genes (STING)-dependent interferon-I-mediated signaling. These findings suggest that SOX2 compromises the immune response in HNSCCs [91,92]. 

## 5. Conclusions

HNSCCs can be characterized by several genomic alterations, involving various pathways. These can widely alter different cellular processes and change the tumor microenvironment, which, in turn, affects cancer progression and immune response. Our data confirm that an increased TMB may be associated with a greater sensitivity to immunotherapeutic agents. Moreover, a distinct signature, in terms of genomic alterations and network connectivity of the circulating soluble adhesion molecules, soluble immuno-checkpoints, cytokines, and chemokines, characterized responder patients, in contrast to non-responder ones and on the basis of clinical outcome. In the future, information from circulating soluble immune checkpoints, along with the complete molecular genomic profile, will need to be interpreted in combination with other factors, including patient clinical features, as well as lifestyle and diet, immunological profile of the tumor microenvironment, metabolic and pharmaco-genomic profile, gut microbiome, and instrumental imaging. This innovative approach to cancer care will allow for patient-specific treatments and new combination strategies, in order to avoid rapid and life-threatening disease progression.

## Figures and Tables

**Table 1:** Clinicopathological characteristics of platinum refractory HPV ^2^ negative R/M HNSCC and previous treatment.

Characteristics	All Patients N 10
Age (years) Median age (range)	66 (46–76)
Gender Male Female	8 2
Baseline PS ^1^ 0 1	2 8
Risk factors Smoking history (SH) Alcohol abuse (AA)	8 3
Tumor Location Oral cavity Oropharynx Larynx	4 1 5
Histology Squamous cell carcinoma	10
Grading 2 3	4 6
HPV ^2^ Positive Negative	0 10
Only recurrent disease Metastatic site ≥ 1 Previous I line (extreme)	1 9 10

^1^ PS: ECOG performance status at baseline, before nivolumab treatment. ^2^ HPV: human papilloma virus.

**Table 2:** Case series: clinicopathological features, molecular characteristics, and outcomes.

Patients	Age	Primary Tumor	PS	Best Response	PFS (Months)	OS (Months)	Early Progression	Patient Status ^1^	Genomic Signature
Case 1	62	Oral cavity	1	PD	2	6	Yes	DOD	TMB-intermediate 8 mut/MB MSI-stable
Case 2	65	Larynx	1	PD	1	21	Yes	ALIVE	TMB 6 muts/MB MSI-stable
Case 3	76	Larynx	1	PD	2	2	Yes	DOD	TMB 6 muts/MB MSI-stable
Case 4	65	Oral cavity	1	PD	2	11	Yes	DOD	TMB 3 muts/MB MSI-stable
Case 5	53	Larynx	1	PD	1	3	Yes	DOD	TMB 4 muts/MB MSI-stable
Case 6	52	Oropharynx	1	SD	5	6	No	DOD	TMB 5 muts/MB MSI-stable
Case 7	63	Oral cavity	1	SD	6	7	No	DOD	TMB 3 muts/MB MSI-stable
Case 8	71(F)	Larynx	0	PR	33	33	No	ALIVE	TMB 30 muts/MB MSI-stable
Case 9	67	Larynx	0	PR	20	39	No	ALIVE	TMB 15 muts/MB MSI-stable
Case 10	46(F)	Oral cavity	1	PR	4	5	No	DOD	TMB 1 muts/MB MSI-stable

^1^ At the last follow-up visit; DOD: death of disease; PFS: progression free survival; OS: overall survival; SD: stable disease; PD: progressive disease; PR: partial response; TMB: tumor mutational burden; MSI: microsatellite instability.

**Table 3:** Genomic profile of patients with R/M HNSCC.

Not Mutated	Case Report									
Mutated	# 1	# 2	# 3	# 4	# 5	# 6	# 7	# 8	# 9	# 10
TMB	8 Muts/Mb	6 Muts/Mb	6 Muts/Mb	3 Muts/Mb	4 Muts/Mb	5 Muts/Mb	3 Muts/Mb	30 Muts/Mb	15 Muts/Mb	1 Muts/Mb
Microsatellite Status	MS-Stable	MS-Stable	MS-Stable	MS-Stable	MS-Stable	MS-Stable	MS-Stable	MS-Stable	MS-Stable	MS-Stable
TP53										
BRCA1										
ARID1A										
CDKN2A/B										
BCL2L1										
NFE2L2										
NOTCH1										
CCND1										
ERBB2										
CDK6										
PIK3CA										
SOX2										
EPHB4										
FGF19										
FGF3										
FGF4										
PRKCI										
TERC										
NOTCH2										
RB1										
SMAD4										
TERT										
FGFR1										
CD22										
NSD3										
PRKCI										
AKT2										
MTAP										
SPEN										
KDM6A										
DNMT3A										
CUL3										
PIK3R1										
BCORL1										
PTEN										
FGF14										
NSD3										
PARK2										
ZNF703										
TET2										
PBRM1										
PIK3C2G										

**Table 4:** Soluble immune molecules showing a statistically significant over-expression in responder patients: characteristics and function.

Soluble Molecules	Class of Molecules	Cell Source	Ligands	Main Function	Type of Action	References
**IL13**	Cytokyne	T CD4 Cells CD8 cells NK B-cells Monocytes Eosinophils Mast cells	IL13Rα1 IL13Rα2	- involved in Th2 immune responses - potentiate expression of adhesion molecules on endothelial cells - activation of magrophages and production of TGFb - key regulator of extracellular matrix	proinflammatory	[47]
**IL17A**	Cytokine	Lymphocytes TCD4 Th17	IL17Rα	- induces IL6 and chemokines production - promotes recruitment of MDSCs into the tumour bed - neutrophil recruitment, anti-microbial molecule and acute phase protein production	proinflammatory	[48]
**TNF alpha**	Cytokine	Macrophages NK T cells	TNFR1 TNFR2	- pro-inflammatory activity - fever - Acute phase response - stimulates cell proliferation and survival	Immune-activation/ pro-inflammatory	[49]
**IL4**	Cytokine	T cells Mast cells	IL4-Rα	- activation of Th2 immune response - Cell growth/activation - B-cell growth factors and stimulate B-cell differentiation.	Pro-inflammatory	[50,51]
**INF alpha**	Cytokine	DC Macrophages NK cells Macrophages Endothelial cells Fibroblasts	INFαR1/2	- NK activation - Cells B proliferation - Possible suppression of Treg cells - Antiviral activity - Enhances MHC expression	Pro-inflammatory immune-activation	[52]
**INF gamma**	Cytokine	Lymphocytes T (th1) CD8 and NK	INFγR1/2	- activation of macrophages - activation of Th1 responses - potential antigen presentation to T lymphocytes - induces apoptosis of tumour cells and reduces VEGF - increases expression of IDO	immunoactivating/ possible immunosuppressive activity)	[53]

DC dendritic cells, IL interleukin, IFN interferon, TNF tumor necrosis factor, NK natural killer, MDSC myeloid derived suppressor cell, TGF trasforming growth factor, VEGF vascular endothelial growth factor, IDO Indoleamine 2,3-dioxygenase.

**Table 5:** Effects of pathways dysregulation in head and neck squamous cell carcinoma.

Gene Alteration	Pathways	Mechanism Effect on Tumor Cells	Effect on Immune System and Response to Immunotherapy
TP53	P53	• dysregulate the transactivation of p53-dependent genes and is predicted to promote tumorigenesis	• Affect the activity of myeloid and T cell • Immune evasion and cancer progression
NOTCH1/2	NOTCH pathway	• proliferation • differentiation • MDSC accumulation • Treatment-resistance • Related to EMT • Depending on cellular context, NOTCH2 can act as either a tumor suppressor or an oncogene	Resistance Immune escape
CDKN2A/B	Rb/INK4/ARF	• dysregulation of the CDK4/6-cyclin-Rb pathway and loss of cell cycle control • Growth suppressive activity of the Rb tumor suppressor	Conflicting evidence regarding the association between CDKN2A genomic alterations and response to ICIs
PIK3CA	PI3K	• Associated with lymph nodes metastasis and tumor stage • Involved in cell growth, proliferation, differentiation, motility, and survival	- uncontrolled activation of the PI3K/AKT pathway induces an immune-tolerant tumor microenvironment - Favorable predictive factor for efficacy of immunotherapy
BRCA1/2	DDR pathway	• Key role in DNA repair process • Genomic instability	- DNA damage triggers immune responses through cell death signals - DDR deficiencies improve tumor recognition of adaptive immune system
ARID1A	Encodes for switch/sucrose nonfermenting (SWI/SNF) chromatin remodeling complexes	• loss of ARID1A may activate the PI3K-AKT pathway • ARID1 is considered a tumor suppression	Immune evasion
SOX2	PI3K-AKT pathway	• SOX2 amplification or overexpression leads to activation of the PI3K-AKT pathway • Promote tumor growth through suppression of stimulation of INF genes (STING)-dependent signaling • Involved in growth, viability, migration, tumorigenicity, and drug resistance of cancer cells	Immune evasion

TP53 tumor protein 53, NOTCH1 Neurogenic locus Notch homolog protein 1, NOTCH 2 Neurogenic locus Notch homolog protein 2, MDSC myeloid derived suppressor cell, EMT epithelial to mesenchymal transition, CDKN2A/B cycline-dependent kinase inhibitor 2A/B, Rb retinoblastoma protein, PI3KCA phosphatidylinositol-4,5-bisphosphate 3-kinase, catalytic subunit alpha, PI3K Phosphoinositide 3-kinase, CDK cyclin-dependent kinase, IC immune checkpoint, PD-L1 programmed death- lingand 1, CDK cycline-dependent kinase, BRCA breast related cancer an-tigens, DDR DNA damage response, ARID1 AT-rich interacting domain-containing protein 1A gene, SOX2 SRY (sex determining region Y)-box 2, AKT protein kinase B.

## Data Availability

Upon reasonable request.

## Supplementary Materials

The following supporting information can be downloaded at: https://www.mdpi.com/article/10.3390/biomedicines10112732/s1, Table S1: This table is composed of three sheets reporting all the connectivity networks at T0 as edge-lists, along with the correlation values and corresponding *p*-values. In particular, the first sheet lists the molecule network connections for responder patients (depicted in Figure 6A); the second sheet lists the molecule network connections for non-responder patients (depicted in Figure 6B); the third sheet lists the molecule network connections shared between responder and non-responder patients. Table S2. This table is composed of two sheets reporting all the connectivity networks at T1 as edge-lists, along with the correlation values and corresponding *p*-values. In particular, the first sheet lists the molecule network connections for responder patients (depicted in Figure 7A); the second sheet lists the molecule network connections for non-responder patients (depicted in Figure 7B). Table S3. This table is composed of four sheets reporting all the connectivity networks at T0 and at T1 as edge-lists, along with the correlation values and corresponding *p*-values. In particular, the first sheet lists the molecule network connections for dead patients at T0; the second sheet lists the molecule network connections for alive patients at T0; the third sheet lists the molecule network connections for dead patients at T1; the fourth sheet lists the molecule network connections for alive patients at T1. Figure S1. Heatmap of molecule expression levels (logarithmic scale) at T0 (A) and T1 (B) across 10 patients, grouped by patients’ status, alive (violet bars) or dead (water blue bars). Colors represent different expression levels, increasing from blue to yellow. Figure S2. (A) Correlation between molecules’ profiles and patient alive status at T1. Statistically significant Spearman correlations (*p*-value ≤0.05) are reported. In the plot, circles are scaled and colored according to the correlation values, increasing from red (negative correlation with the alive status) to blue (positive correlation with the alive status). Molecules are grouped and ordered according to the functional group reported in the legend. (B) Boxplot of sE-selectin molecule expression level (logarithmic scale) in three alive patients (violet box) and seven dead ones (water blue box) at T1. *p*-value (*p*) was obtained by performing a Mann–Whitney test for unpaired samples and was equal to 0.06. Figure S3. Connectivity map between molecules in alive (A) and dead (B) patients at T0. Statistically significant Spearman correlations (*p*-value ≤ 0.05) are reported. In the plot, circles are scaled and colored according to the correlation values, increasing from red (negative correlation) to blue (positive correlation). Molecules are grouped and ordered according to the functional group reported in the legend. Figure S4. Connectivity map between molecules in alive (A) and dead (B) patients at T1. Statistically significant Spearman correlations (*p*-value ≤ 0.05) are reported. In the plot, circles are scaled and colored according to the correlation values, increasing from red (negative correlation) to blue (positive correlation). Molecules are grouped and ordered according to the functional group reported in the legend.
