# Supplementary material for: Genomic and Immune Approach in Platinum Refractory HPV-Negative Head and Neck Squamous Cell Carcinoma Patients Treated with Immunotherapy: A Novel Combined Profile
Source: Biomedicines. 2022 Oct 28;10(11):2732. doi: 10.3390/biomedicines10112732 (PMC9687656; doi:10.3390/biomedicines10112732)
Supplement: Supplementary file 1 [file biomedicines-10-02732-s001.zip › biomedicines-1901565-Supplementary.pdf]

Supplementary File

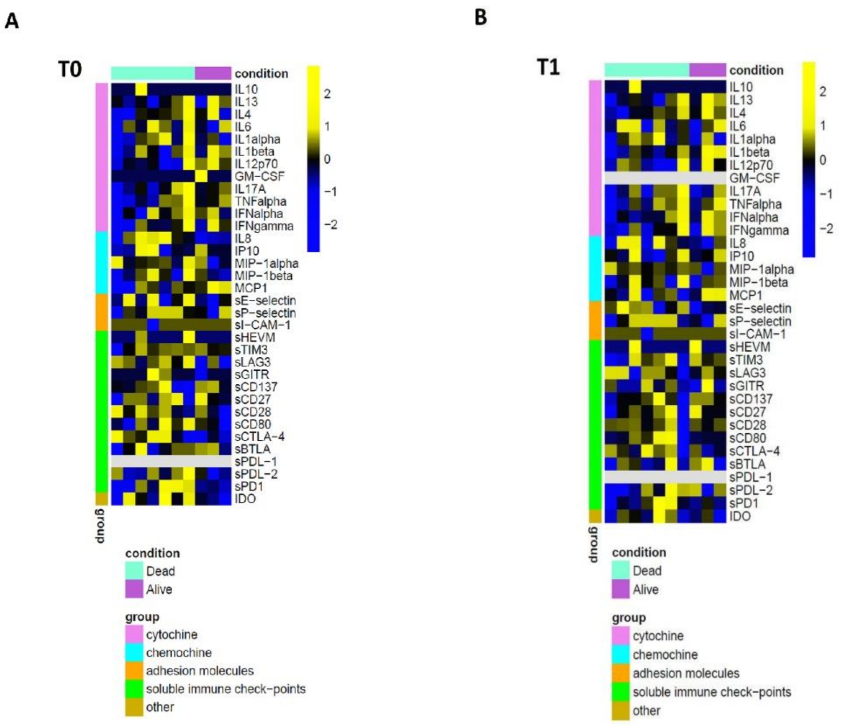

Suppelentary Figure S1. Heatmap of molecule expression levels (logarithmic scale) at T0 (A) and T1 (B) across 10 patients, grouped by patients status alive (violet bars) and dead (water blue bars). Colors represent different expression levels, increasing from blue to yellow.

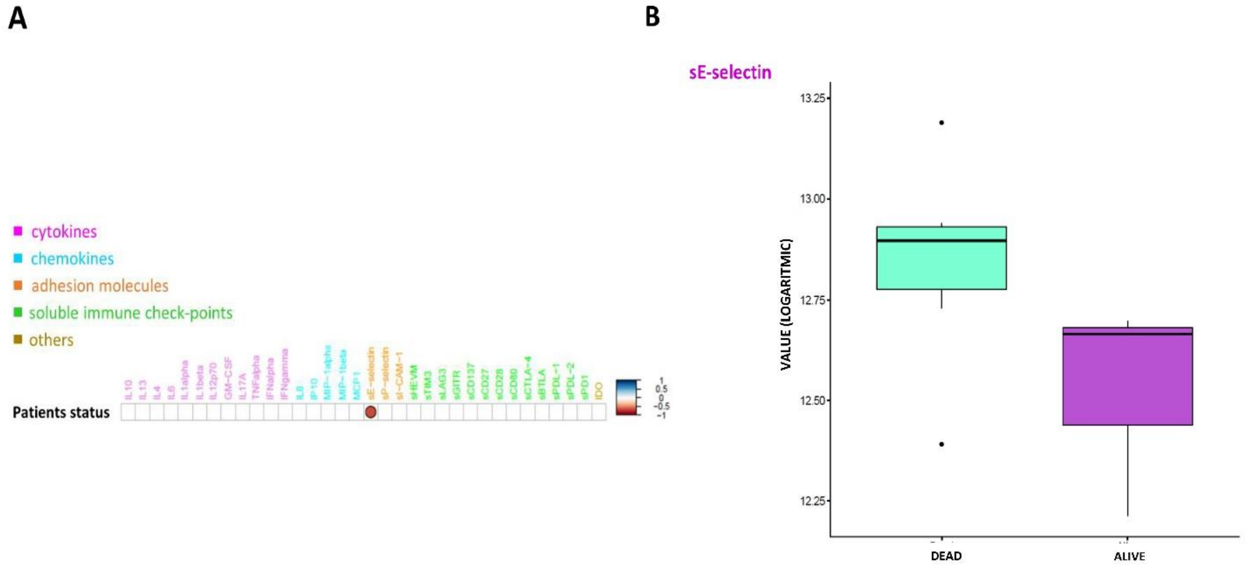

Suppelentary Figure S2. **(A)** Correlation between molecules' profiles and patient alive status at T1. Statistically significant Spearman correlations (p-value ≤0.05) are reported. In the plot, circles are scaled and coloured according to the correlation values, increasing from red (negative correlation with the alive status) to blue (positive correlation with the alive status). Molecules are grouped and ordered according to the functional group reported in the legend. **(B)** Boxplot of sE-selectin molecule expression level (logarithmic scale) in 3 alive patients (violet box) and 7 dead ones (water blue box) at T1. P-value (p) was obtained by performing a Mann-Whitney test for unpaired samples and was equal to 0.06.

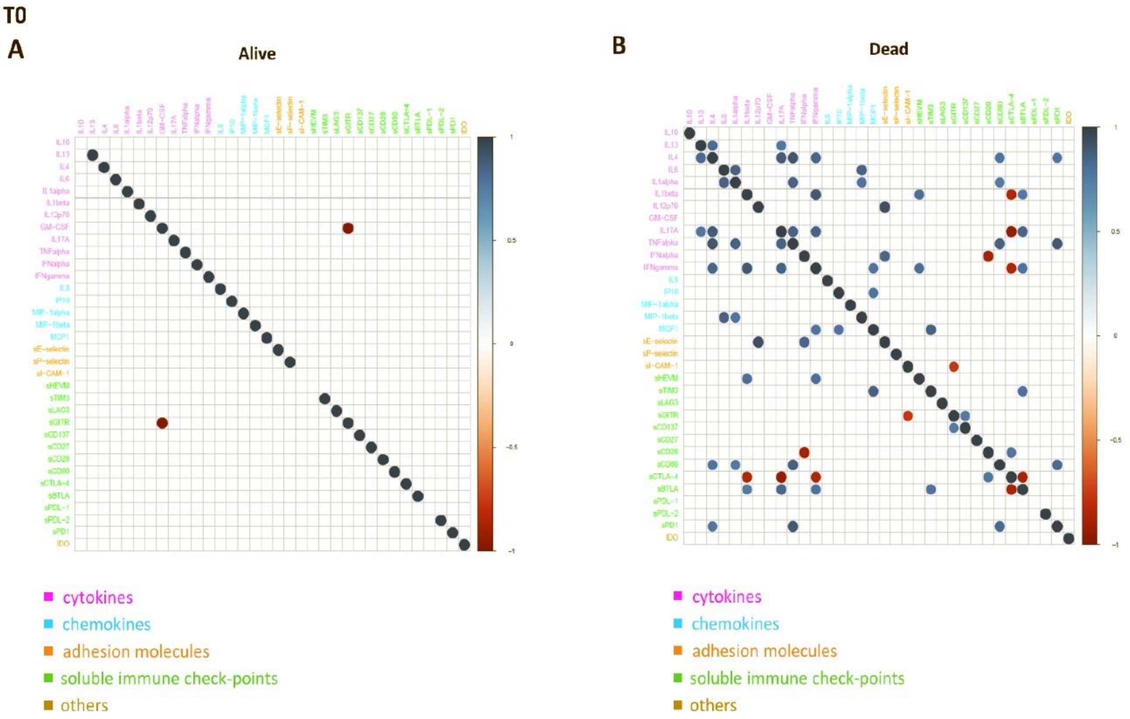

Supplementary Figure S3. Connectivity map between molecules in alive (A) and dead (B) patients at T0. Statistically significant Spearman correlations (p-value ≤ 0.05) are reported. In the plot, circles are scaled and coloured according to the correlation values, increasing from red (negative correlation) to blue (positive correlation). Molecules are grouped and ordered according to the functional group reported in the legend.

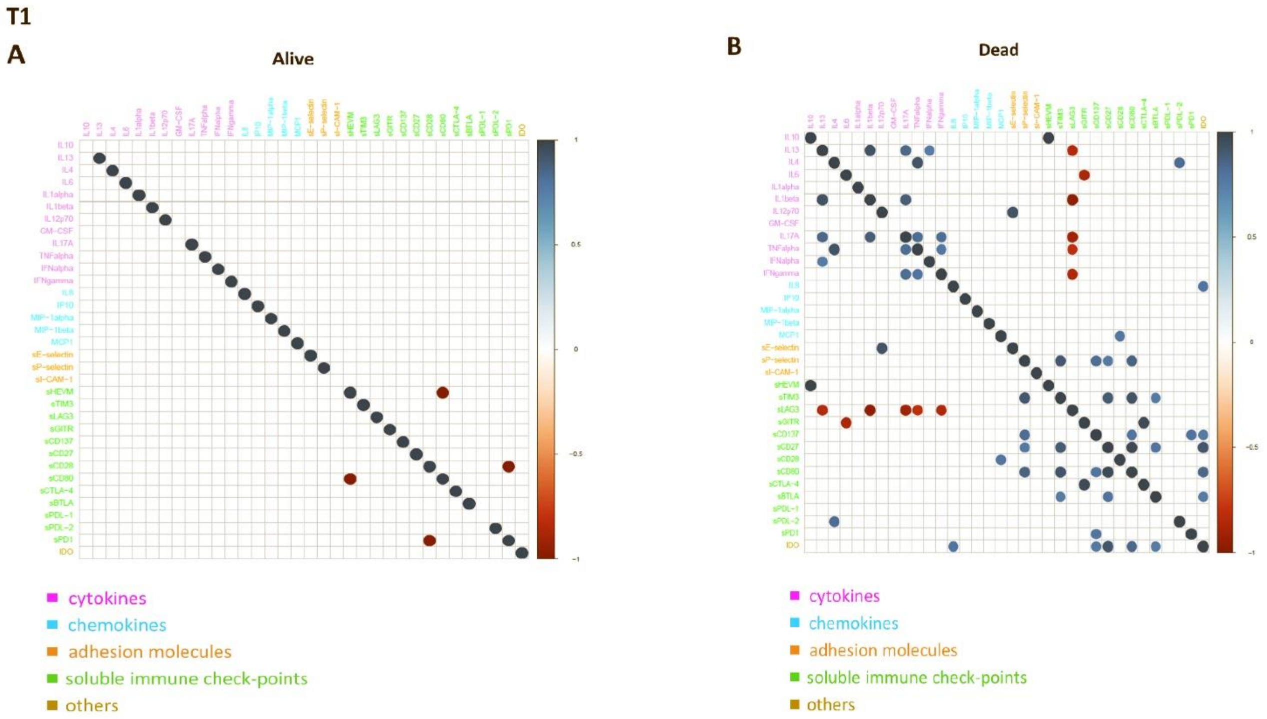

Supplementary Figure S4. Connectivity map between molecules in alive (A) and dead (B) patients at T1. Statistically significant Spearman correlations (p-value ≤ 0.05) are reported. In the plot, circles are scaled and coloured according to the correlation values, increasing from red (negative correlation) to blue (positive correlation). Molecules are grouped and ordered according to the functional group reported in the legend.

***Supplementary Table S3.xlsx:** This table is composed of 4 sheets reporting all the connectivity networks at T0 and at T1 as edge-lists, along with the correlation values and corresponding p-values. In particular, the first sheet lists the molecule network connections for dead patients at T0; the second sheet lists the molecule network connections for alive patients at T0; the third sheet lists the molecule network connections for dead patients at T1; the fourth sheet lists the molecule network connections for alive patients at T1.
